# Characterizing Warthin-Like Mucoepidermoid Carcinoma: Clinicopathologic Features and MAML2 Rearrangements in 14 Latin American Cases

**DOI:** 10.1007/s12105-026-01896-1

**Published:** 2026-05-29

**Authors:** Ricardo Anderson de Oliveira Vasconcelos, Isaac Santos Araújo, Luiz Miguel Ferreira, João Paulo Gonçalves de Paiva, Igor Lima Fernandes, Lucas Faria Abrahao-Machado, Claudia Haydee Sarai Caro-Sánchez, Ana María Cano-Valdez, Adalberto Mosqueda-Taylor, Ciro Dantas Soares

**Affiliations:** 1 Oral Diagnosis Department, Piracicaba Dental School, University of Campinas (UNICAMP), Avenida Limeira 901, Areião, Piracicaba, São Paulo 13414-900 Brazil; 2 Private Pathology Service, Getúlio Sales Diagnósticos, GSD, Natal, Rio Grande do Norte Brazil; 3 Bacchi Pathology Laboratory, Botucatu, São Paulo Brazil; 4 National Cancer Institute, Mexico City, Mexico; 5 Health Care Department, Universidad Autónoma Metropolitana, Mexico City, Mexico

**Keywords:** Warthin-like mucoepidermoid carcinoma, Salivary gland, Mucoepidermoid carcinoma, Warthin tumor

## Abstract

**Purpose:**

Warthin-like mucoepidermoid carcinoma (WL-MEC) is a low-grade variant of mucoepidermoid carcinoma (MEC), characterized by a multicystic growth pattern and dense lymphoid stroma, histologically mimicking Warthin tumor (WT). Few cases have been reported, limiting understanding of this variant. We aimed to provide a comprehensive clinical, histopathologic, and molecular analysis of new WL-MEC cases.

**Methods and Results:**

Fourteen WL-MEC cases from Brazil, Guatemala, and Mexico were analyzed. Clinicopathologic features (patient demographics, tumor location, AFIP grading, follow-up, recurrence), histological characteristics (architectural patterns, inflammatory infiltrate, epithelial components, invasion), and immunohistochemical profiles (CK5/6, p63, p40, CK7, CK14, Ki67, HER2) were assessed. *MAML2* generearrangements and *HER2* amplification were evaluated by fluorescence in situ hybridization (FISH). All 14 cases showed *MAML2* rearrangement (100%), confirming the diagnosis, while *HER2* amplification was absent in all 9 cases tested.

**Conclusions:**

This study provides new insights into the clinicopathological and molecular features of WL-MEC, confirming its typical presentation (parotid gland, middle-aged females), low-grade behavior, and favorable prognosis after surgical excision. Detection of *MAML2* rearrangement is a valuable diagnostic tool for distinguishing WL-MEC from WT.

## Introduction

Warthin-like mucoepidermoid carcinoma (WL-MEC) is a recently described variant of mucoepidermoid carcinoma (MEC) with characteristic histological features and low-grade behavior [1]. The term “WL-MEC” was designated due to its prominent multicystic growth pattern and dense lymphoid stroma, which histologically mimics conventional Warthin tumor (WT) [1, 2]. This striking morphological similarity between WL-MEC and WT underscores the critical importance of thorough diagnostic evaluation, particularly given their prognostic implications [3].

Clinically, WL-MEC typically presents as asymptomatic nodular masses, predominantly occurring in the parotid gland, while a subset of cases affects the minor salivary glands of the palate. Based on previous reports this variant exhibits a female predilection and occurs across a wide age range, showing peak prevalence during the sixth decade of life [3].

Histologically, WL-MEC shows cystic and solid nests of mucous, intermediate, and squamoid/epidermoid cells, frequent oncocytic change, set within a dense lymphoid stroma [2–5]. At the molecular level, WL-MEC characteristically harbors *MAML2* rearrangements, most commonly *CRTC1*::*MAML2* as consequence of t(11;19)(q21;p13) and less commonly *CRTC3*::*MAML2* as consequence of t(11;15)(q21;q26) [6]. This genetic signature serves as a key diagnostic marker, enabling reliable distinction from WT with metaplastic changes [1, 3]. The detection of *MAML2* rearrangements through fluorescence in situ hybridization (FISH) and reverse transcription polymerase chain reaction (RT-PCR) has become a key diagnostic tool for accurate diagnosis [3]. While *HER2* gene amplification has been reported in conventional MEC, predominantly in high-grade tumors [7], specific data for *HER2* in WL-MEC are limited to date.

Our recent systematic review [3] highlighted both the exceptional rarity of reported WL-MEC cases and the critical need for larger studies to better characterize its clinicopathological features. In response to this gap of knowledge, we present a well-documented Latin American case series study of 14 new WL-MEC cases, significantly expanding the current understanding of this variant.

## Materials and methods

### Study Design, Case Selection and Data Collection

This study employed a retrospective clinicopathological design. To identify potential cases of Warthin-like Mucoepidermoid Carcinoma (WLMEC), a broad search was initially conducted in the pathology archives of institutions in Brazil, Guatemala, and Mexico. The search terms included ‘Warthin tumor with dysplasia,’ ‘atypical Warthin tumor,’ ‘infarcted Warthin tumor,’ and ‘Mucoepidermoid carcinoma with lymphoid stroma.’ All retrieved cases underwent blind re-evaluation by five pathologists. Inclusion criteria were strictly defined as tumors exhibiting the classic WLMEC morphology (cystic architecture lined by oncocytic/squamoid epithelium with a prominent lymphoid component) confirmed by AFIP grading standards. Cases with equivocal morphology were only included upon confirmation of *MAML2* rearrangement by FISH. Based on this selection process, fourteen cases were included.

Ethical approval was obtained from the relevant institutional research committee (Protocol number 76629323.4.0000.5418), adhering to the Helsinki Declaration principles. Informed consent for participation and publication was obtained from all individuals whose data were included. Clinical data were collected from patient records, including age, sex, tumor location, surgical management, and follow-up information (duration, recurrence, and status).

### Histopathological Evaluation

Formalin-fixed, paraffin-embedded (FFPE) tissue sections were stained with hematoxylin and eosin (H&E) for morphological assessment. Histological features evaluated included architectural pattern (multicystic, unicystic or solid), presence of conventional MEC areas, germinal center formation, intraluminal mucous secretions (confirmed with PAS staining), composition of inflammatory infiltrate (lymphocytes, macrophages, plasma cells) based on the morphology and qualitatively assessed, cell type components (oncocytic, intermediate, mucous, epidermoid), epithelial layering, cellular and nuclear pleomorphism, perineural invasion, vascular invasion, and invasion into adjacent tissues. Tumors were graded according to the Armed Forces Institute of Pathology (AFIP) grading system. The cases were reviewed by five study pathologists, and the histologic features were recorded.

### Immunohistochemistry (IHC)

Immunohistochemical staining was performed on 4-µm thick sections obtained from formalin-fixed, paraffin-embedded (FFPE) tissue blocks. All reactions were conducted using a fully automated Ventana Benchmark XT™ platform (Ventana Medical Systems, Tucson, AZ, USA) according to the manufacturer’s protocols. The panel of primary antibodies utilized, including specific clones and sources, is detailed in Table 1.

**Table 1:** Immunohistochemical markers employed in WL-MEC cases

Antibody	Clone	Dilution
CK5/6	D5/16B4	Prediluted
CK7	OV-TL 12/30	Prediluted
CK14	LL 002	Prediluted
p40	BC28	Prediluted
p63	4A4	Prediluted
EMA	E29	1:100
S100	Polyclonal	Prediluted
GFAP	GA5	1:100
Calponin	Polyclonal	Prediluted
Ki67	MIB-1	1:100
HER2	VENTANA anti-HER2/neu (4B5)	Prediluted

For the assessment of HER2 protein expression, the pre-diluted VENTANA anti-HER2/neu (4B5) Rabbit Monoclonal Primary antibody was used. Initial evaluation and scoring of HER2 IHC expression followed the criteria outlined in the Human Epidermal Growth Factor Receptor 2 (HER2) Breast Testing Guideline Update (ASCO/CAP) [8].

For all other primary antibodies listed in Table 1, immunoreactivity was assessed qualitatively, classifying expression simply as positive or negative based on the presence or absence of specific staining within the neoplastic cells. Stained slides were evaluated manually using conventional light microscopy. Additionally, whole-slide images (WSI) were generated and reviewed using Aperio ImageScope software (Leica Biosystems, Wetzlar, Germany) to confirm findings.

### Molecular Analysis

Molecular testing was conducted using Fluorescence in situ hybridization (FISH) on formalin-fixed paraffin-embedded (FFPE) sections to detect *MAML2* rearrangements and *HER2* amplification. Four-micron-thick FFPE sections were pretreated with 0.01 M Sodium Citrate solution (LK-110 C) for 15 min, followed by incubation in 2 × SSC and subsequent pretreatment with Kreatech solution B (#LK-100 C). Enzymatic digestion was performed using Proteinase K (Sigma Aldrich) for 5 min. Sections were denatured at 75 °C for 5 min, hybridized at 37 °C for 16 h, and counterstained with DAPI.

#### *MAML*2 Rearrangement FISH Analysis

FISH utilized a commercially available *MAML2* dual-color break-apart probe (Z-2014-200, Zytovision, Germany). Analysis under fluorescence microscopy identified cells with two fused signals (one orange, one green) as normal. Cells exhibiting *MAML2* gene rearrangements showed one normal fusion signal alongside one separate orange and one separate green signal. A known *MAML2*-rearranged mucoepidermoid carcinoma served as a positive control, and normal salivary tissue served as a negative control.

#### *HER2* Amplification

*HER2* FISH was conducted using the automated Thermobrite Elite™ platform (Leica Biosystems, Buffalo Grove, IL) with a Kreatech™ *ERBB2* (17q12) / SE 17 dual-color probe (*ERBB2* labeled with PlatinumBright™550, SE 17 labeled with PlatinumBright™495). Scoring was performed on a minimum of 120 nuclei. Amplification was defined according to ASCO/CAP recommendations: a *HER2* (red)/CEP17 (green) signal ratio ≥ 2.0 and ≥ 4.0 *HER2* signals per nuclei (group 1); ≥6.0 *HER2* signals per nuclei if *HER2* (red)/CEP17 (green) signal ratio < 2.0 (if IHC 2 + or 3+)(group 3); ≥4.0 and < 6.0 *HER2* signals per nuclei with *HER2* (red)/CEP17 (green) signal ratio < 2.0 if IHC is 3+ (group 4); and < 4.0 *HER2* signals per nuclei with *HER2* (red)/CEP17 (green) signal ratio ≥ 2.0 if IHC is 3+ (group 2). Breast carcinoma served as a positive control, and normal salivary tissue served as a negative control [9].

## Results

Our study identified 14 WL-MEC cases originating from Brazil (*n* = 10), Guatemala (*n* = 3), and Mexico (*n* = 1). Patient ages ranged from 38 to 74 years (mean: 57.4 ± 10.7; median: 59 years), with female patients (mean age: 57.8 years) outnumbering males (mean age: 56.6 years) by a 2.25:1 ratio (9 females [64.3%] vs. 5 males [35.7%]). All cases in this series (*n* = 14/ 100%) occurred in the parotid gland and were managed with parotidectomy. According to the Armed Forces Institute of Pathology (AFIP) grading system, most tumors were classified as low-grade (*n* = 13/ 92.9%), with a single case designated as intermediate-grade (*n* = 1/ 7.1%) (Table 2).

**Table 2:** Clinical and demographic features of WL-MEC cases included in this case series study

Case	Country	Age	Sex	Site	Grade - AFIP	Treatment	Recurrence	Follow-up	Time (months)
#1	Mexico	74	F	PG	Low	Parotidectomy	Unknown	Unknown	Unknown
#2	Guatemala	42	M	PG	Low	Parotidectomy	No	Alive	14
#3	Guatemala	58	F	PG	Intermediate	Parotidectomy	No	Alive	39
#4	Guatemala	55	F	PG	Low	Parotidectomy	No	Alive	53
#5	Brazil	65	F	PG	Low	Parotidectomy	No	Alive	19
#6	Brazil	60	F	PG	Low	Parotidectomy	Yes	Alive	22
#7	Brazil	63	F	PG	Low	Parotidectomy	No	Alive	10
#8	Brazil	63	M	PG	Low	Parotidectomy	No	Alive	48
#9	Brazil	53	M	PG	Low	Parotidectomy	Unknown	Unknown	Unknown
#10	Brazil	38	F	PG	Low	Parotidectomy	Unknown	Unknown	Unknown
#11	Brazil	71	M	PG	Low	Parotidectomy	Unknown	Unknown	Unknown
#12	Brazil	48	F	PG	Low	Parotidectomy	Unknown	Unknown	Unknown
#13	Brazil	46	F	PG	Low	Parotidectomy	No	Alive	31
#14	Brazil	71	M	PG	Low	Parotidectomy	Yes	Alive	13

AFIP, armed forces institute of pathology; F, female; M, male; PG, parotid gland

Among patients with available follow-up data (*n* = 9), recurrence was observed in 2 cases (22.2%). The follow-up duration for these patients ranged from 10 to 53 months, with a mean follow-up time of 26.8 ± 15.6 months and a median follow-up time of 22 months. Notably, all 9 patients were alive at their last documented follow-up (Table 2).

**Fig. 1:**
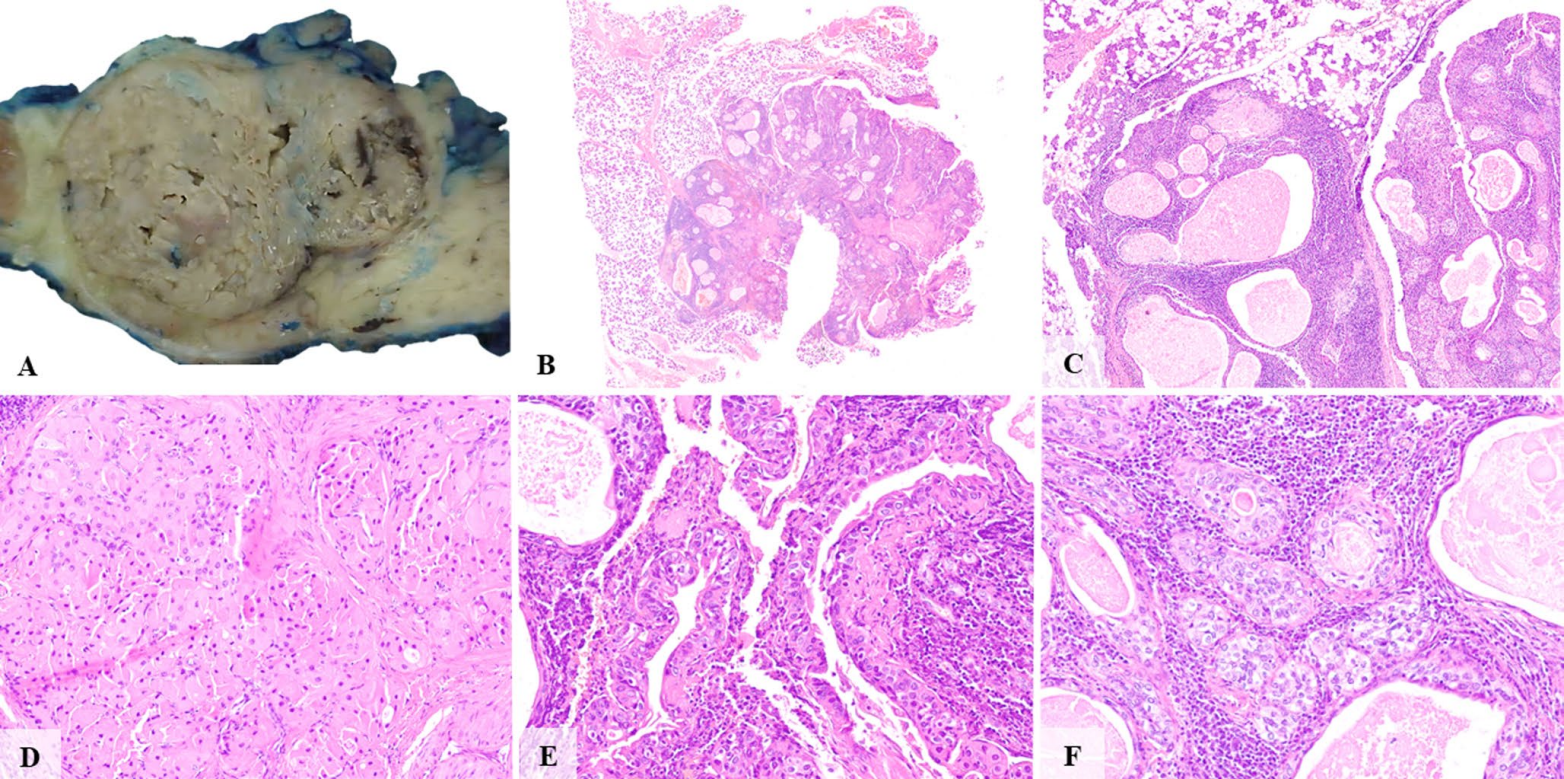
**A** Macroscopic appearance of a WL-MEC showing a lobulated, greyish, partially cystic lesion. **B** A well-circumscribed yet locally infiltrative neoplasm affecting the parotid gland parenchyma. **C** The tumor exhibited a multicystic architecture and was associated with a dense lymphocytic inflammatory infiltrate. **D** Solid proliferations of oncocytic cells, reminiscent of the epithelial component in some epithelial-rich Warthin tumors. **E** Cystic spaces are predominantly lined by a disorganized, multilayered oncocytic epithelium. **F** Higher magnification highlights a focal area of conventional mucoepidermoid differentiation, characterized by mucous and intermediate cells adjacent to the oncocytic proliferation

Histologically, the architectural pattern was predominantly multicystic (*n* = 13/ 92.9%), with a single case being unicystic (7.1%). All 14 cases exhibited germinal center formation (100%), PAS-positive intraluminal secretions, and a mixed inflammatory infiltrate comprising lymphocytes, macrophages, and plasma cells. In all cases, the inflammatory infiltrate was predominantly composed of lymphocytes; however, in three cases (21.4%), macrophages accounted for approximately 40% of the infiltrate. Conventional MEC areas were identified in 2 of 14 cases (14.3%) (Figs. 1 and 3, and Table 3).

**Fig. 2:**
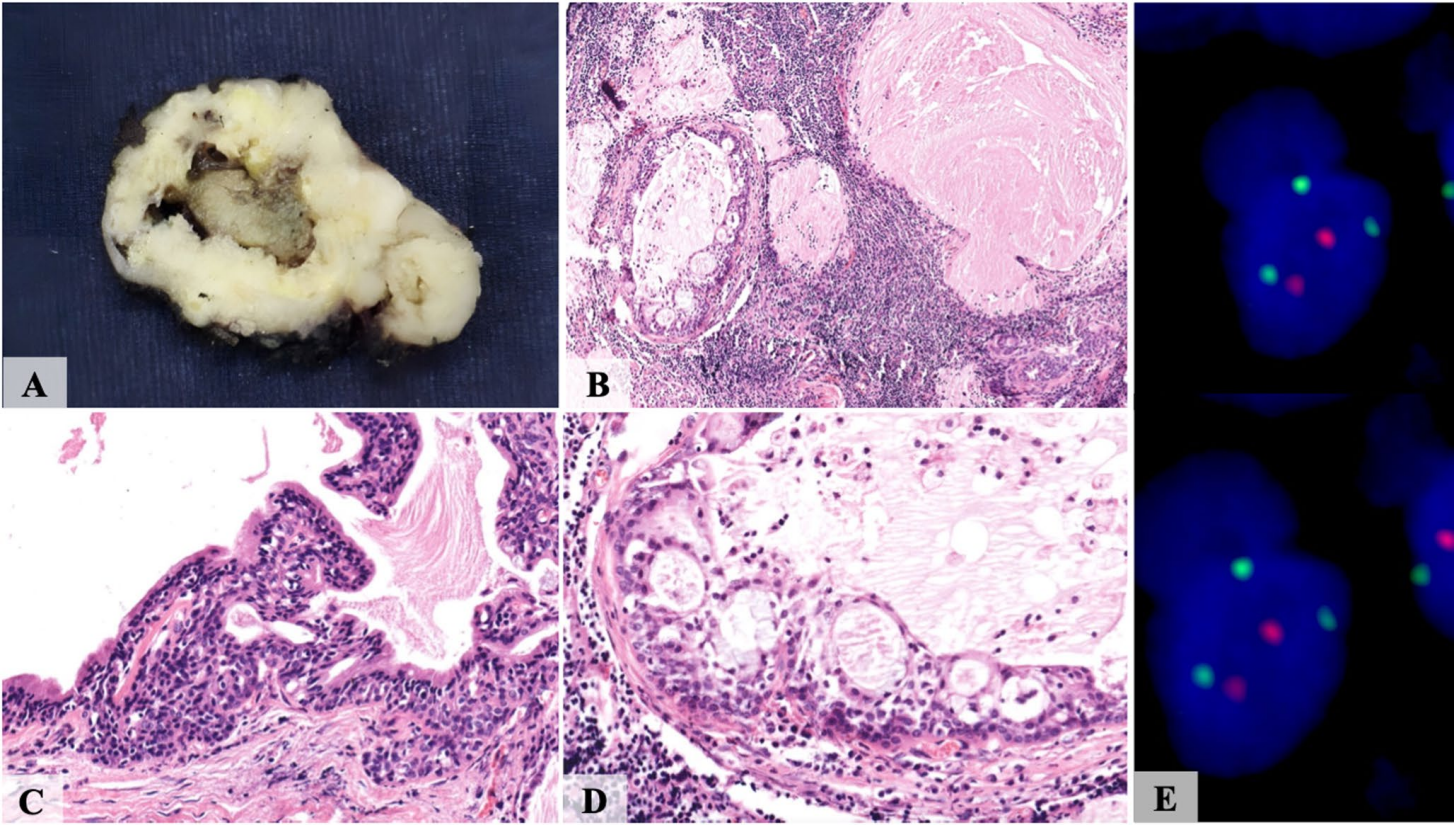
**A** A cystic lesion with lobulated borders. **B** The WL-MEC was composed of multiple cysts embedded in a dense lymphocytic inflammatory infiltrate. **C** Most cysts were delineated by a two-layered oncocytic epithelium that was occasionally disorganized. **D** The cystic epithelium was composed of epidermoid, intermediate, and mucous cells arranged in microcysts, which secreted an eosinophilic material. **E** *MAML2* dual-color break-apart FISH demonstrating signal rearrangement

**Table 3:** Histopathological features of WL-MEC cases included in this case series

Case	Loculation pattern	Inflammatory infiltrate*	Germinal center	Secretions	Oncocytic cells	Two-layered epithelium	Intermediate cells	Mucous cells	Conventional MEC areas	Pleomorphism	VI	PNI	Invasive component
#1	Multicystic	Lym 60/ MØ 40	Y	Y, PAS+	Y	Y	Y	Y	N	Mild	N	N	Y
#2	Multicystic	Lym	Y	Y, PAS+	Y	Y	Y	Y	N	Mild	N	N	Y
#3	Multicystic	Lym	Y	Y, PAS+	Y	Y	Y	Y	N	No	N	N	Y
#4	Multicystic	Lym 60/ MØ 40	Y	Y, PAS+	Y	N	Y	Y	Y	Moderate	N	N	Y
#5	Multicystic	Lym	Y	Y, PAS+	N	Y	Y	Y	N	Mild	N	N	N
#6	Multicystic	Lym	Y	Y, PAS+	N	Y	Y	Y	N	Mild	N	N	N
#7	Multicystic	Lym	Y	Y, PAS+	Y	Y	Y	Y	Y	Moderate	N	N	Y
#8	Multicystic	Lym	Y	Y, PAS+	Y	Y	Y	Y	N	Moderate	N	N	Y
#9	Unicystic	Lym	Y	Y, PAS+	Y	Y	Y	Y	N	Mild	N	N	Y
#10	Multicystic	Lym 60/ MØ 40	Y	Y, PAS+	N	Y	Y	Y	N	Mild	N	N	Y
#11	Multicystic	Lym	Y	Y, PAS+	Y	Y	Y	Y	N	Mild	N	N	Y
#12	Multicystic	Lym	Y	Y, PAS+	N	Y	Y	Y	N	Moderate	N	N	Y
#13	Multicystic	Lym	Y	Y, PAS+	Y	Y	Y	Y	N	Mild	N	N	Y
#14	Multicystic	Lym	Y	Y, PAS+	Y	Y	Y	Y	N	Mild	N	N	Y

Y: yes, N: no, PAS: Periodic acid- Schiff, +: positive, MEC: Mucoepidermoid carcinoma, NO: none, Lym: lymphocytes, MØ: macrophages, VI: vascular invasion, PNI: perineural invasion. *Lymphocytes, macrophages, and plasma cells were observed in all cases. However, only the most prevalent cell type was mentioned

**Fig. 3:**
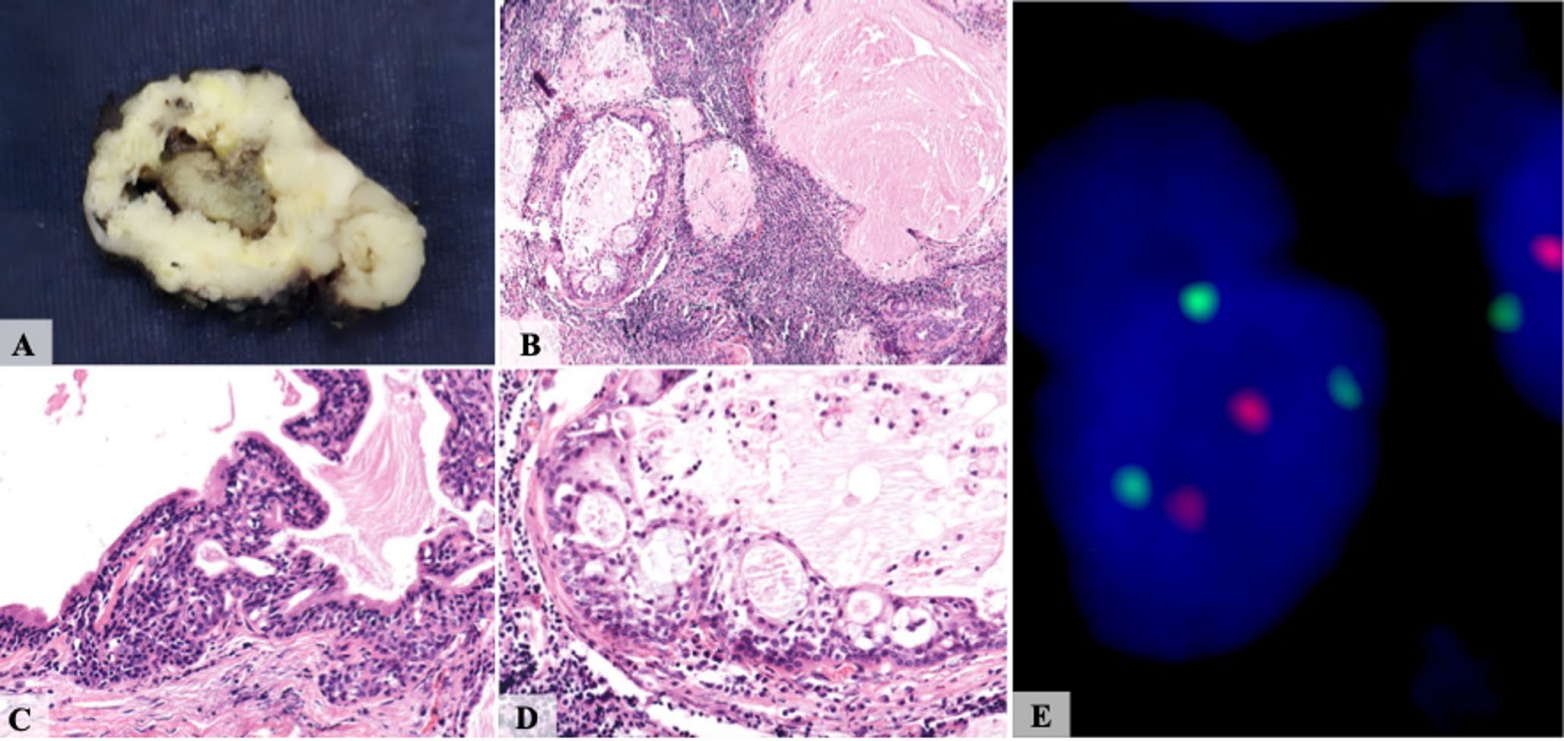
Immunohistochemical features of WL-MEC. The lesions consistently exhibited positivity for **A** CK7. **B** CK14. **C** p63, and **D** p40

The cell type components included oncocytic cells (*n* = 10/ 71.4%), intermediate cells (*n* = 14/ 100%), and mucous cells (*n* = 14/ 100%). Notably, four of the 14 cases lacked identifiable oncocytic cells, a finding consistent with the initial description of WL-MEC, in which oncocytic change was not regarded as a defining feature [2]. A characteristic two-layered epithelial architecture was observed in most cases (*n* = 13/ 92.9%). Cellular and nuclear pleomorphism was absent in 1 case (7.1%), while being present in a mild degree (*n* = 9/ 64.3%) and moderate degree (*n* = 4/ 28.6%) in the remaining cases. None of the 14 cases exhibited perineural or vascular invasion. An invasive component was observed in 11 cases (78.6%) (Figs. 1 and 3, and Table 3).

All WL-MEC cases (*n* = 14/ 100%) demonstrated strong and diffuse expression of CK5/6, proteins p63 and p40, CK7, and CK14. S100 protein positivity was identified in a few cases (*n* = 4/ 28.6%), while all cases uniformly lacked GFAP and calponin immunoreactivity (*n* = 14/ 100%). The proliferation index measured by Ki67 ranged from 4.2% to 15.1% (mean 8.2%) (Fig. 3; Table 4). HER2 expression by IHC was consistently negative (score 0) in all cases evaluated. *HER2* FISH was negative in all cases investigated (*n* = 9/ 100%). *MAML2* rearrangements by FISH were uniformly positive in all 14 cases (100%) (Fig. 3; Table 4).

**Table 4:** Immunohistochemical markers and molecular tests performed in WL-MEC cases included in this case series

Case		CK5/6	p63	p40	EMA	S100	CK-7	CK-14	GFAP	Calponin	HER2 Score (IHC)	KI67* (%)	*MAML2* rearrangment (FISH)	HER2 (FISH)
#1		Pos	Pos	Pos	Pos	Neg	Pos	Pos	Neg	Neg	0	9.7	Pos	Neg
#2		Pos	Pos	Pos	Pos	Neg	Pos	Pos	Neg	Neg	0	5.3	Pos	Neg
#3		Pos	Pos	Pos	Pos	Neg	Pos	Pos	Neg	Neg	N/D	11.1	Pos	N/D
#4		Pos	Pos	Pos	Pos	Pos	Pos	Pos	Neg	Neg	N/D	5.8	Pos	N/D
#5		Pos	Pos	Pos	Pos	Pos	Pos	Pos	Neg	Neg	0	4.2	Pos	Neg
#6		Pos	Pos	Pos	Pos	Pos	Pos	Pos	Neg	Neg	0	7.9	Pos	Neg
#7		Pos	Pos	Pos	Pos	Neg	Pos	Pos	Neg	Neg	N/D	4.9	Pos	N/D
#8		Pos	Pos	Pos	Pos	Neg	Pos	Pos	Neg	Neg	0	10.1	Pos	Neg
#9		Pos	Pos	Pos	Pos	Pos	Pos	Pos	Neg	Neg	0	10.5	Pos	Neg
#10		Pos	Pos	Pos	Pos	Neg	Pos	Pos	Neg	Neg	N/D	5.6	Pos	N/D
#11		Pos	Pos	Pos	Pos	Neg	Pos	Pos	Neg	Neg	0	10.1	Pos	Neg
#12		Pos	Pos	Pos	Pos	Neg	Pos	Pos	Neg	Neg	0	9.1	Pos	Neg
#13		Pos	Pos	Pos	Pos	Neg	Pos	Pos	Neg	Neg	N/D	5.6	Pos	N/D
#14		Pos	Pos	Pos	Pos	Neg	Pos	Pos	Neg	Neg	0	15.1	Pos	Neg
	Pos: positive, Neg: negative, N/D: not determined. * Ki67 was measured only in tumor cells, excluding areas of inflammatory infiltrate.													

## Discussion

The accurate diagnosis of WL-MEC is critical due to its histopathological overlap with WT, a benign tumor with distinct prognostic and therapeutic implications [10, 11]. This distinction directly impacts clinical management, as WL-MEC, despite its indolent behavior, represents a malignancy requiring comprehensive oncological intervention [2]. WL-MEC remains an uncommon entity, with the current understanding of its clinical, morphological, and molecular characteristics predominantly derived from isolated case reports and small series [3]. This study provides a substantial contribution to the literature by presenting the largest Latin American cohort to date, including 14 clinicopathologically and molecularly confirmed WL-MEC cases.

Our findings revealed a higher prevalence in female patients, aligning with the findings of our published systematic review, which reported a female predominance of 66.67% and a female-to-male ratio of 2:1 [3]. This epidemiological profile notably diverges from WT, which classically exhibits male predominance and is associated with smoking habits [12]. Additionally, the mean patient age in this series (57.4 years) was significantly higher than that reported for conventional mucoepidermoid carcinoma (48.8 years), suggesting a distinct later-onset age distribution pattern [13]. In our cohort, all WL-MECs localized exclusively in the parotid gland, consistent with previous reports demonstrating a strong predilection for this site [3]. This anatomical distribution parallels that of WT, which similarly favors the parotid gland [12]. However, unlike WT, WL-MEC has been documented in other salivary glands. Data on clinical symptoms were not available in our sample. Nevertheless, the literature suggests that WL-MEC commonly presents as an asymptomatic, slow-growing nodule, similar to other salivary gland neoplasms [3, 13].

Histologically, WL-MECs exhibit a remarkably consistent profile across reported cases. The tumors typically show a multicystic architecture accompanied by a prominent lymphoid stroma, often with germinal center formation. The epithelial component consists of a variable mixture of mucous, oncocytic, intermediate, and epidermoid cells [5, 14]. Although a bilayered epithelium is a defining architectural feature of Warthin tumor, prior studies have emphasized that the bilayered appearance in WL-MEC is usually incomplete, irregular, and frequently disrupted by an admixture of mucous, intermediate, and epidermoid cells [2]. This pattern contrasts with the uniform, stable bilayer characteristic of Warthin tumor. Consistent with this description, most cases in our series (13/14) showed a two-layered appearance; however, the bilayer was not continuous and was often altered by the neoplastic epithelial components typical of WL-MEC. This variability aligns with the original report by Ishibashi et al. [2], who emphasized that the neoplastic epithelium of WL-MEC is “not typically oncocytic”, distinguishing it from oncocytic MEC with lymphoid stroma. In our series, 4 of 14 cases (28.6%) exhibited no oncocytic component, reinforcing that this feature, although common, is not mandatory for diagnosis.

The identification of areas morphologically resembling conventional MEC within a Warthin-like background appears to be a relatively frequent observation [3]. In our study, these areas were observed in two cases and were very focal. Most of the tumor usually has an appearance similar to WT, and these foci of conventional MEC remain limited and can be difficult to recognize [14]. This aligns with Bishop (2022) [15], who emphasized that even a very small focus of conventional MEC in a well-sampled cystic salivary gland neoplasm is highly informative for establishing the correct diagnosis. Additionally, although the presence of cytologic atypia and mucous cells is a relevant feature in distinguishing WL-MEC from Warthin tumor, it is important to acknowledge that WT may occasionally display mucinous metaplasia. However, in WT such foci are generally limited and lack atypia, in contrast to the mucous differentiation integrated with intermediate and epidermoid cells that is typical of WL-MEC [3, 16, 17].

Our immunohistochemical analysis revealed consistent, strong, and diffuse expression of epithelial markers CK5/6, proteins p63 and p40, CK7, and CK14 in all cases, supporting the squamous and ductal epithelial phenotype of WL-MEC. This diffuse p63/p40 pattern is a key diagnostic clue, contrasting with the basal-restricted pattern characteristic of Warthin tumor (WT) and papillary cystadenoma, as well as the discordant expression seen in other oncocytic lesions [18–20]. It is noteworthy that although WT may occasionally exhibit mucinous metaplasia, such foci lack the accompanying architectural complexity (e.g., intermediate and epidermoid cell components) and more substantial cytologic atypia that are defining features of WL-MEC. Myoepithelial markers, GFAP and calponin, were consistently negative in WL-MEC, helping to exclude pleomorphic adenoma and other lesions with prominent myoepithelial components [19]. While assessment of proliferative activity by Ki67 is not an independent diagnostic or prognostic marker in WL-MEC, it may provide complementary data, particularly in distinguishing it from WT, which characteristically demonstrates lower proliferative activity [21]. In our series, the Ki67 index had a mean value of 8.2%, consistent with the low-grade clinical behavior typically described for this variant [22].

The diagnosis of MEC is generally established based on histological features, and in most cases, ancillary diagnostic tests are unnecessary. Nevertheless, molecular assessment of *MAML2* rearrangement becomes particularly relevant in tumors with atypical morphology that depart from the classic MEC pattern and overlaps with other neoplastic entities. This is especially applicable to MEC, which has a predominance of oncocytic cells, known as the oncocytic variant. In this context, the detection of *MAML2* rearrangements plays a crucial role in differentiating MEC from other oncocytic neoplasms, such as oncocytoma and oncocytic carcinoma [23].

In the context of WL-MEC, suspicion may initially be raised based on histopathological features. However, molecular confirmation has become a valuable tool for establishing a definitive diagnosis in diagnostically challenging cases. Molecular methods for diagnostic confirmation using FISH, capable of detecting rearrangements involving the *MAML2* gene (particularly the *CRTC1::MAML2* fusion), have become the tool used to confirm the lesion [24]. The *MAML2* rearrangement was confirmed by FISH in 100% of the cases in our series, as well as in the study by Bishop et al., [14], reinforcing its role as a possible key diagnostic marker for WL-MEC and supporting diagnostic accuracy in clinical practice.

This finding is particularly noteworthy when compared to the broader spectrum of MEC, where *MAML2* rearrangement sensitivity ranges from 66% to 100%, depending on histological grade and tumor variant [25]. While *MAML2*-negative cases have been documented in conventional MEC, particularly in high-grade tumors where positivity rates drop to 16-28.6% [26, 27], and in oncocytic variants where approximately 29% were *MAML2*-negative [23], the WL-MEC variant appears, to represent a distinct molecular subset characterized by a consistently high rate of positivity for *MAML2* rearrangements [3]. Wang et al. [5] corroborated this observation in their study of 29 WL-MEC cases, where all cases demonstrated *MAML2* rearrangement, and a comprehensive Polish study examining 114 WT found that while none of the benign WT harbored *MAML2* rearrangements, all 22 reported WL-MEC cases in the literature were *MAML2*-positive [24]. This molecular consistency suggests that *MAML2* rearrangement may be a defining feature of the WL-MEC phenotype, distinguishing it from other MEC variants. This molecular signature is crucial for resolving diagnostic ambiguities, especially in distinguishing WL-MEC from WT, which is characterized by the absence of molecular alterations involving the *MAML2* gene [14]. In addition to *MAML2* status, other molecular markers may aid in the differential diagnosis. The consistent negativity of *HER2* amplification in our series (0/9 cases tested) aligns with the typically low-grade nature of WL-MEC and helps distinguish it from potentially HER2-positive salivary gland tumors, such as certain high-grade salivary duct carcinomas [28].

Accurate diagnosis of WL-MEC is critical for appropriate clinical management [29]. This low-grade malignancy requires complete surgical excision with clear margins, coupled with long-term monitoring due to its potential for recurrence and lymph node metastasis [30]. In contrast to WT, which may be managed with superficial parotidectomy or periodic surveillance, the surgical approach for WL-MEC should prioritize complete resection with negative margins. This may be achieved by either superficial or total parotidectomy, depending on tumor size, location, and depth of parenchymal involvement [31, 32]. All patients in this series underwent parotidectomy, with a recurrence rate of 22.2%, which is slightly higher than that reported in our systematic review on WL-MEC (8.82%) [3]. This difference may be explained by the fact that WL-MEC closely mimics WT both clinically and macroscopically, which can lead to initially conservative surgical approaches, such as enucleation or resections with close margins [3]. In the setting of a malignant neoplasm, technically free but close margins may allow for microscopic residual disease, potentially contributing to local recurrence [31]. Notably, all patients were alive at the last follow-up, highlighting the favorable prognosis of WL-MEC. No patient in this series required adjuvant therapy, but in selected scenarios, such as positive margins or high-grade lesions, cervical dissection and radiotherapy may be indicated [32].

Our study offers notable strengths, including a relatively robust cohort (*n* = 14) for this exceptionally rare neoplasm and comprehensive clinicopathological characterization, supported by molecular confirmation of *MAML2* rearrangements. However, several limitations warrant acknowledgement. The retrospective study design may introduce inherent selection bias and constrain generalizability, while precluding advanced statistical analyses. Although our sample size is substantial for a rare tumor, it remains limited for multivariate or survival analyses. Furthermore, the heterogeneous and relatively limited follow-up duration in some cases precludes definitive assessment of long-term outcomes, particularly recurrence dynamics and survival trends. A systematic absence of granular clinical data, including patient ethnicity, symptomatology, precise tumor dimensions, and radiologic features, further limited clinicopathological correlations. Additionally, while five pathologists reviewed all cases, a formal centralized pathology review process was not implemented. Future multicentric collaborative efforts with extended surveillance periods and larger cohorts are essential to validate our observations and elucidate the biological behavior of WL-MEC.

In summary, WL-MEC is a rare MEC variant that typically presents as an asymptomatic parotid gland nodule, shows a female predilection, and most often affects patients in the sixth decade of life (mean age 57.4 years). Histologically characterized by low-grade morphology, multicystic architecture and lymphocytic-rich stroma. In cases where diagnosis is difficult based solely on morphological and immunohistochemical characteristics, molecular detection of *MAML2* gene rearrangements can serve as a complementary diagnostic tool. Surgical excision represents the gold standard management approach, typically yielding favorable outcomes with low metastatic potential and recurrence rates in adequately resected cases.

## Data Availability

No datasets were generated or analysed during the current study.
